# Comparative analysis of machine learning techniques on the BraTS dataset for brain tumor classification

**DOI:** 10.3389/fonc.2025.1596718

**Published:** 2025-09-15

**Authors:** Shuping Wang, Min Li

**Affiliations:** ^1^ Information Statistics Center, Hubei Cancer Hospital, Tongji Medical College, Huazhong University of Science and Technology, Wuhan, China; ^2^ School of Computer Science and Technology, Hubei Business College, Wuhan, China

**Keywords:** brain tumor, classification accuracy, machine learning techniques, high-grade gliomas (HGG), comparative study

## Abstract

**Introduction:**

Accurate classification of brain tumors from MRI scans is a critical task for improving patient outcomes. Machine learning (ML) and deep learning (DL) methods have shown promise in this domain, but their relative performance remains unclear.

**Methods:**

This study evaluates several ML and DL techniques using the BraTS 2024 dataset. The models assessed include traditional algorithms such as Random Forest and advanced deep learning architectures including Simple CNN, VGG16, VGG19, ResNet50, Inception-ResNetV2, and EfficientNet. Preprocessing strategies were applied to optimize model performance.

**Results:**

The Random Forest classifier achieved the highest accuracy of 87%, outperforming all deep learning models, which achieved accuracy in the range of 47% to 70%. This indicates that traditional ML approaches can sometimes surpass state-of-the-art DL methods in tumor classification tasks.

**Discussion:**

The findings highlight the importance of model selection and parameter tuning in automated brain tumor diagnosis. While deep learning models are generally considered standard for image analysis, Random Forest demonstrated superior performance in this context. This underscores the need for fine-grained consideration of dataset characteristics, computational resources, and diagnostic requirements.

**Conclusion:**

The study shows that carefully selected and optimized ML approaches can improve tumor classification and support more accurate and efficient diagnostic systems for brain tumor patients.

## 1 Introduction

**Figure 1:**
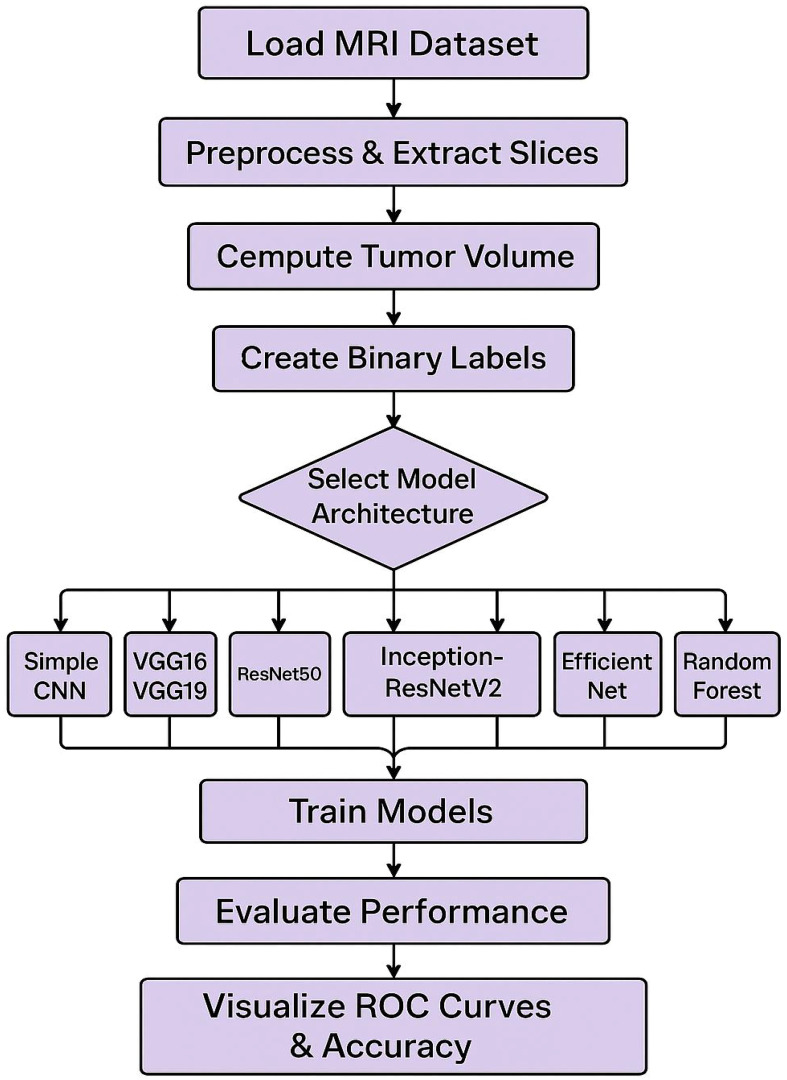
Workflow diagram for tumor detection.

The combination of deep learning and machine learning techniques has resulted in advancement of brain tumor segmentation and classification. The application of these approaches has been a revolution in the field of medical imaging primarily by automating the traditional manually driven processes and accuracy of the tumor identification and classification. As demonstrated by U-Net and Convolutional Neural Networks (CNNs) type of deep learning models, tumor regions in 2D MRI images can be accurately delineated with high Dice similarity coefficients for tumor regions (1, 2). In addition to this, mathematical and machine learning methods such as thresholding, K-Means clustering, and CNN are employed to solve the segmentation problem as an optimization problem by using extracted features to precisely pinpoint tumor edges (3). The segmentation process has been further refined by such other advanced image processing techniques as noise reduction, image enhancement and wavelet analysis (4). As for classification, CNNs have been used effectively for differentiating various tumor types with high classification (1, 5). Support Vector Machines (SVMs) and Random Forests have also been used for tumor classification (**Figure 1**) by finding best separating hyperplanes or decision trees grown in the feature space (extracted using other set of features) (3). New and more sophisticated neural network architectures, such as NeuraClassNet (6) and MDCNet (7) have reached 99.67% accuracies via novel optimization techniques and multi-view analyses. However, these advancements do not guarantee solution, mainly due to data and model complexity. By using the traditional image processing and the contemporary computational intelligence methods, robust solutions for diverse imaging modalities can be achieved by addressing these constraints, thereby enhancing clinical applicability (8).

The steps include loading the MRI dataset, preprocessing the data, computing tumor volume, creating binary labels, and splitting the data into training and testing sets. The model architecture is selected from various options, including Simple CNN, VGG16/VGG19, ResNet50, Inception-ResNetV2, Efficient Net, and Random Forest. The models are trained and subsequently evaluated for performance, with the results visualized through ROC curves and accuracy metrics. Convolutional Neural Networks (CNNs) have become essential for brain tumor classification because they perform automatic feature extraction from medical imaging data. Pre-trained CNN models have received validation from multiple research studies for their effectiveness in this area. In brain tumor classification medical imaging applications VGG-16 demonstrated its effectiveness by reaching a high 98% accuracy rate according to studies by Vellanki et al., 2024 (9) and Vetrivelan et al., 2024 (10). The ResNet architecture, especially ResNet50, produced strong results with validation accuracies reaching up to 89.47% according to Muftic et al. and Padmakala & Maheswari both in 2024. The EfficientNetB0 and EfficientNetB3 models shows effectiveness by achieving high accuracy levels of 98.36% and 99.44%, respectively (11–13). DenseNet models have superior performance by achieving validation accuracies up to 95% (14). The pretrained models that use transfer learning mechanism has the capability to reuse features from large datasets thereby providing significant benefits for medical imaging applications that has limited dataset availability. Hybrid and ensemble learning approaches are used to further enhance the classification accuracy by combining the advantages of multiple models. As an example, the PDSCNN-RRELM combines a lightweight parallel depthwise separable convolutional neural network (PDSCNN) with a hybrid ridge regression extreme learning machine (RRELM) and achieves a great average precision of 99.35% (15). The custom four residual deep learning architecture (4RDL-DCNN) optimized by particle swarm optimization achieved high effectiveness with the accuracy of 98.60% (16) was another noteworthy model. Additionally, a classification accuracy of 95% (17) was achieved on a ViT and EfficientNet-V2 ensemble combined through a genetic algorithm. Ensemble methods demonstrated consistently better results than individual models, benefitting from the strength of combining different architectures to obtain better results. Improvement of model performance and building trust rely heavily on explain ability and optimization techniques. The accuracies achieved for various datasets (11) with the integration of the Quantum Genetic Algorithm (QGA) with EfficientNetB0 are 98.36 and 98.25. In a similar manner, the Particle Swarm Optimization (PSO) algorithm is introduced to optimize the feature extraction process in the custom 4RDL-DCNN model obtaining an accuracy of 98.60% (16). Moreover, weights in the combined system of Vision Transformers (ViT) and EfficientNet-V2 models are optimized using the Genetic Algorithm (GA) to achieve 95% classification accuracy (17). The optimization approaches also improve the feature selection and model weight adjustments to yield improved classification performance. Although deep learning models dominate the field, conventional machine learning (ML) techniques have shown potential in brain tumor classification. The Gaussian Process Classifier (GPC), combined with principal component analysis (PCA), has exhibited notable improvements in accuracy, precision, recall, and F1-score (18). Support Vector Machines (SVMs) have also been utilized alongside feature selection methods like the Gini index and mutual information, yielding competitive results (18, 19). Despite their inherent advantages, traditional machine learning (ML) models generally underperform compared to deep learning architectures in image-based classification tasks.

In medical applications, explain ability is extremely important in order to achieve clinical trust in model decisions. To clarify its decision making process, the PDSCNN-RRELM model has been incorporated with Shapley Additive Explanations (SHAP) (15). Moreover, Gradient-weighted Class Activation Mapping (Grad-CAM) has been employed to visualize the decision making of deep learning models in order to increase their interpretability (20). These approaches make deep learning models in clinical environments transparent and reliable. Significant improvement of model performance was achieved through the application of transfer learning and advanced data preprocessing techniques. Pre trained models such as VGG16, Resnet50, EfficientNetB3 were fine-tuned on brain tumor datasets to achieve the state of the art results (12, 13). Additionally, data augmentation techniques such as sparse auto encoder based augmentation and contrast limited adaptive histogram equalization (CLAHE) (16, 20) were used to enhance the diversity of the dataset and improve model generalization. Different performance metrics such as accuracy, precision, recall and F1 score have been used to evaluate various models effectiveness. Vellanki et al. made noteworthy findings, namely that VGG-16 has achieved 98% accuracy in brain tumor classification while Gencer & Gencer obtained 98.36% accuracy over traditional methods with EfficientNetB0. The denseNet has shown good generalization to a validation accuracy of 95% (14). In addition, the accuracies of ensemble models using multiple deep learning architectures (21) can reach up to 99.43%. There has been progress, but there are still a lot of obstacles. It was observed that models such as EfficientNetB3 and VGG-19, when trained on a limited amount of data, exhibited signs of overfitting (12, 13). In order to train more complex models, larger datasets are required (12, 13). Furthermore, most of the models have been tested on the standard datasets, which makes it important to evaluate them further in real clinical settings (14, 22). Other issues should be tackled in future studies and new architectures and optimization methods explored to improve model accuracy and dependability.

The BraTS 2024 database (32) contains T1w, T1w contrast enhancement (T1c), T2w, and FLAIR sequence types, each offering distinct information for imaging various aspects of tumor morphology and structure (23). This research concentrated on utilizing a subset of these modalities, specifically contrast-enhanced T1 (T1c), T2w, and T2-FLAIR (or T2w) images for tumor classification. Additionally, the corresponding segmentation masks were employed to extract quantitative measurements (such as tumor size) used to create binary labels. Patients with tumor volumes exceeding the median were classified as having high tumor burden, while those below the median were categorized as having low tumor burden. For decades, deep learning has the potential to solve the complex patterns in medical images. The architectures used in this study include VGG16 (24), VGG19 (25), ResNet50 (26), Inception-ResNetV2 (27), and EfficientNet (28), all of which are known to achieve state-of-the-art performance in various image classification tasks. Even in this case, classical machine learning techniques, e.g., Random Forests, are very competitive (and in combination with PCA robust feature extraction, which is in particular useful when there is a lack of data and/or the features are very discriminative) (29, 30). Despite numerous studies utilizing these methods, no comprehensive evaluation of this nature on the BraTS 2024 dataset has been reported in the existing literature. This gap is filled by evaluating several state of the art deep learning models as well as Random Forest classifier on PCA reduced features. Additionally, the models were compared in terms of accuracy, loss, and confusion matrices, and the results were visualized at both individual and aggregate levels (40–42).

This research has three main goals. First, it evaluates how different deep learning models and a traditional Random Forest classifier perform when applied to the BraTS 2024 dataset. Second, it examines how various data preprocessing techniques and labeling approaches affect model effectiveness. Lastly, this investigation provides valuable insights and suggestions for enhancing brain tumor classification methods. The findings are intended to inform future studies and practical applications of automated brain tumor detection and analysis.

## 2 Materials and methods

This section describes the BraTS 2024 dataset and outlines the preprocessing pipeline implemented to prepare the data for model training. The focus was on leveraging multimodal MRI scans, extracting relevant features, and generating labels for binary classification.

### 2.1 Dataset

The BraTS 2024 dataset provides a comprehensive collection of multimodal MRI scans of patients with brain tumors https://arxiv.org/abs/2405.18368 (32). The dataset was obtained from a Kaggle repository and organized into patient-specific folders. For example, a typical folder (for example, BraTS-GLI-02632-102) contains files named according to the following pattern:

- BraTS-GLI-02632-102-t1c.nii
- BraTS-GLI-02632-102-t2w.nii
- BraTS-GLI-02632-102-t2f.nii
- BraTS-GLI-02632-102-seg.nii

This hierarchical structure facilitates individual patient-level processing, ensuring that each modality and its corresponding segmentation mask are correctly associated with each other. Harmonizing data for training requires a preprocessing step. For the data preprocessing pipeline, nibabel library was used to load the NIfTI files. Middle slices were extracted from each modality (T1c, T2w, and T2-FLAIR) assuming that they represent the tumor region. Finally, each extracted slice was resized to 128×128 pixels using the resize function from skimage. Transform and normalized to the [0,1] range to reduce variability across scans. A stack of 3 channel image was formed for each patient by stacking the processed slices from the three modalities along the channel dimension. Then, a segmentation mask was used to compute the tumor volume by counting nonzero voxels, which served as a quantitative measure used to develop classification labels. As there is no explicit clinical labels in the dataset, the binary classification labels were computed based on the tumor volume. More specifically, for all patients the median tumor volume was calculated and if a tumor volume exceeded the median, 1 was assigned as its label (indicative of a high tumor burden). Patients with volumes above the median were labelled with a value of 1, indicating a high tumor burden, and patients with volumes below the median were given a label of 0. It also leads to a balanced binary labelling scheme for the classification task. The data were preprocessed and labeled, and the data were partitioned into training and testing subsets by 80/20 ratio. The partition was done at patient level so that there are no patient data in both the training and the testing sets. This approach helps in keeping the independence of the evaluation data and gives a fair estimate of the model performance. The following section details how the BraTS 2024 dataset was prepared for classification. The first step of preprocessing made sure that the imaging data were the same size and intensity, and the second step ensured that the label generation step allowed for a clear definition of classes in terms of the tumor burden. These procedures were used to guide subsequent model training and evaluation as laid out in the next sections. Each patient folder in the dataset contained several NIfTI files corresponding to different imaging modalities, including:

- T1c (Contrast-Enhanced T1): Highlights regions with blood–brain barrier disruption.
- T2w (T2-Weighted): Provides a high signal intensity for fluid regions.
- T2-FLAIR (Fluid-Attenuated Inversion Recovery): Suppresses cerebrospinal fluid signals to better visualize the lesions.
- Segmentation Masks: Contain expert annotations delineating the tumor regions.

The T1c, T2w, and T2-FLAIR modalities were utilized to construct a 3-channel input image for each patient, while the segmentation mask was used to derive quantitative measures, such as tumor volume.

Primarily one needs to load the dataset and perform pre-processing on the data and utilize PCA for dimensional reduction. The information undergoes division into training batches and testing batches. The Random Forest model receives its training through application of the training data. The testing data receives predictions from the model and its performance undergoes evaluation. The visual representation includes the ROC curve.

### 2.2 Methodology

**Figure 2:**
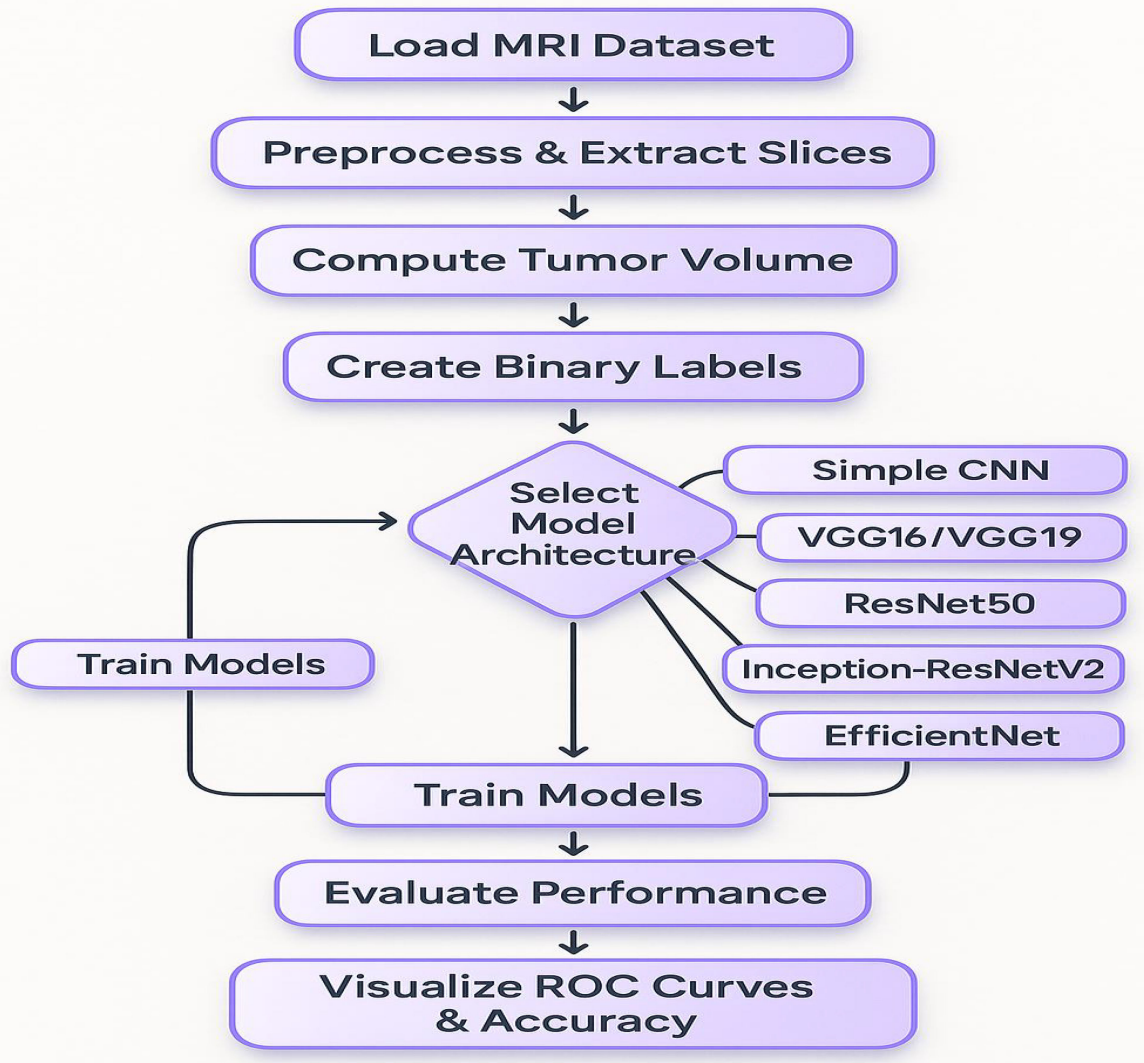
A flowchart presents the complete procedure for data analysis.

This section outlines the overall experimental design, including the models evaluated, training procedures, and the evaluation metrics and visualizations used to compare performance (**Figure 2**). To capture the intricate patterns, present in brain MRI data, several state-of-the-art deep learning architectures were implemented using transfer learning. The foundational baseline model employed was a Simple CNN, representing a custom-designed convolutional neural network. Subsequently, the VGG16 architecture, consisting of 16 layers, was utilized due to its suitability for transfer learning by freezing the convolutional base and appending custom fully connected layers. This was followed by the use of VGG19, which extends the architecture to 19 layers to enable the extraction of more abstract features. Additionally, ResNet50, a 50-layer residual network with shortcut connections, was incorporated to facilitate the training of deeper models. Inception modules and the residual connection-based advanced hybrid architecture, InceptionResNetV2, were introduced to capture multi-scale features. Lastly, EfficientNet was employed, which balances model depth, width, and resolution through compound scaling, representing a modern and efficient network design. In neural models, an increase in the number of layers generally results in a higher number of trainable parameters (weights and biases), allowing the network to model more complex relationships. However, deeper architectures are more prone to overfitting, particularly when the dataset is limited, and thus require additional regularization and tuning strategies to generalize effectively (38, 39). A Random Forest classifier is the classical machine learning approach which, in addition to deep learning models, was used for classification. Then, for this method, image detecting features were extracted through flattening the pixel intensities and followed by the dimensionality reduction method of Principal Component Analysis (PCA). Under conditions where the dataset size was limited, this classical technique acted as a benchmark for the deep learning models.

In this study on brain tumor classification using the BraTS 2024 dataset, a Random Forest classifier was employed to distinguish between cases of high and low tumor volumes. Prior to classification, each patient’s multi-modal brain MRI comprising t1c, t2w, and t2f sequences is preprocessed and flattened into a high-dimensional feature vector *x*. To reduce dimensionality and mitigate overfitting, Primary Component Analysis (PCA) is applied to project these feature vectors onto a lower-dimensional space.

This transformation is mathematically expressed in Equation 1 as:


(1) $$z=w^{T}(x-x)$$

Where *x* represents the mean of the training data and *W* is the projection matrix containing the top principal components. The transformed feature vector *z* thus captures the most significant variance in the data while discarding redundant information.

The Random Forest classifier then operates on these PCA-reduced features. Let (*z*) denote the prediction made by the *k* − *th* decision tree in the ensemble for the transformed input *z*. In a binary classification setting (with class labels 0 for low tumor volume and 1 for high tumor volume), the final prediction y^  is obtained through a majority voting scheme as presented in Equation 2:


(2) y^ = modeT1z, T2z, …, TKz

This process can also be formulated in Equation 3 as:


(3) y^=arg maxc∑k=1K1{Tk(z)=c}

Where $\left\{\text{it}\right\}1\left\{=c\right\}\left\{/\text{it}\right\}\;$ is an indicator function that equals 1 if the *k* − *th* tree predicts class *c* and 0 otherwise.

Alternatively, if each tree provides a probabilistic estimate *pk* for class *c*, the ensemble’s class probability is calculated as the average of these estimates as shown in Equation 4:


(4) p^ c∣x= 1k∑k=1Kpk c∣x

The final class label is then determined by selecting the class with the highest average probability as presented in Equation 5:


(5) y =argmaxc p^ (c∣x)

#### 2.2.1 Data preprocessing pipeline

In the context of the BraTS2024 dataset, this approach leverages the multi-modal imaging data by first condensing it via PCA and subsequently classifying patients based on tumor volume. The middle slice from each modality (T1c, T2w, and T2-FLAIR) was extracted and resized to 128×128 pixels. The three modalities were stacked to form a three-channel image, with each channel corresponding to a specific modality. All images were normalized to the [0, 1] range to maintain consistency across models.

#### 2.2.2 Training configuration

The dataset was split into training and testing sets using an 80/20 ratio, ensuring no patient appeared in both sets. Data augmentation techniques (rotation, flip, and scaling) were applied to enhance generalization. The Adam optimizer was employed with learning rates (1e-4 to 1e-3), and Categorical Cross-Entropy served as the loss function for binary classification with one-hot encoded labels. Training used a batch size of 8 and initially spanned five epochs, extended as needed for improved performance.

#### 2.2.3 Model architectures and training

For deep learning models, the convolutional bases of pretrained models were frozen during initial experiments, with options for unfreezing and fine-tuning in future trials. For the Random Forest approach, images were flattened, and PCA reduced feature dimensionality (minimum of 50 components). The Random Forest classifier was trained with 100 estimators and default parameters as a classical benchmark.

#### 2.2.4 Performance evaluation

Model performance was assessed through:

- Training/validation accuracy and loss curves across epochs
- Confusion matrices analyzing true/false positives/negatives
- ROC curve analysis with AUC values
- Aggregate plots comparing final accuracies and losses
- Grid layouts of all confusion matrices

This comprehensive evaluation framework enabled robust comparison of both deep learning and classical machine learning approaches on the BraTS 2024 dataset.

## 3 Results

This section presents the experimental results of the BraTS dataset 2024 used in this study. Deep learning models and classical Random Forest classifier are evaluated for comparison. The performances of both individual and aggregated models are visualized in terms of accuracy, loss and confusion matrices. The experiments are evaluated using the BraTS 2024 dataset. The data in the dataset is preprocessed for each patient by extracting the middle slices of three imaging modalities (T1c, T2w, T2-FLAIR) and normalizing them. To create binary labels from the tumor volume computed based on the segmentation masks, the median volume was chosen as the threshold (**Table 1** *)*. The following accuracy results were obtained for the test set:

**Table 1:** Obtained results of accuracy.

S. No	Model	Accuracy
1	Random Forest (with PCA features)	87.5%
2	Simple CNN	70.0%
3	VGG16	67.5%
4	VGG19	62.5%
5	Inception-ResNetV2	60.0%
6	ResNet50	47.5%
7	Efficient Net	47.5%

**Figure 3:**
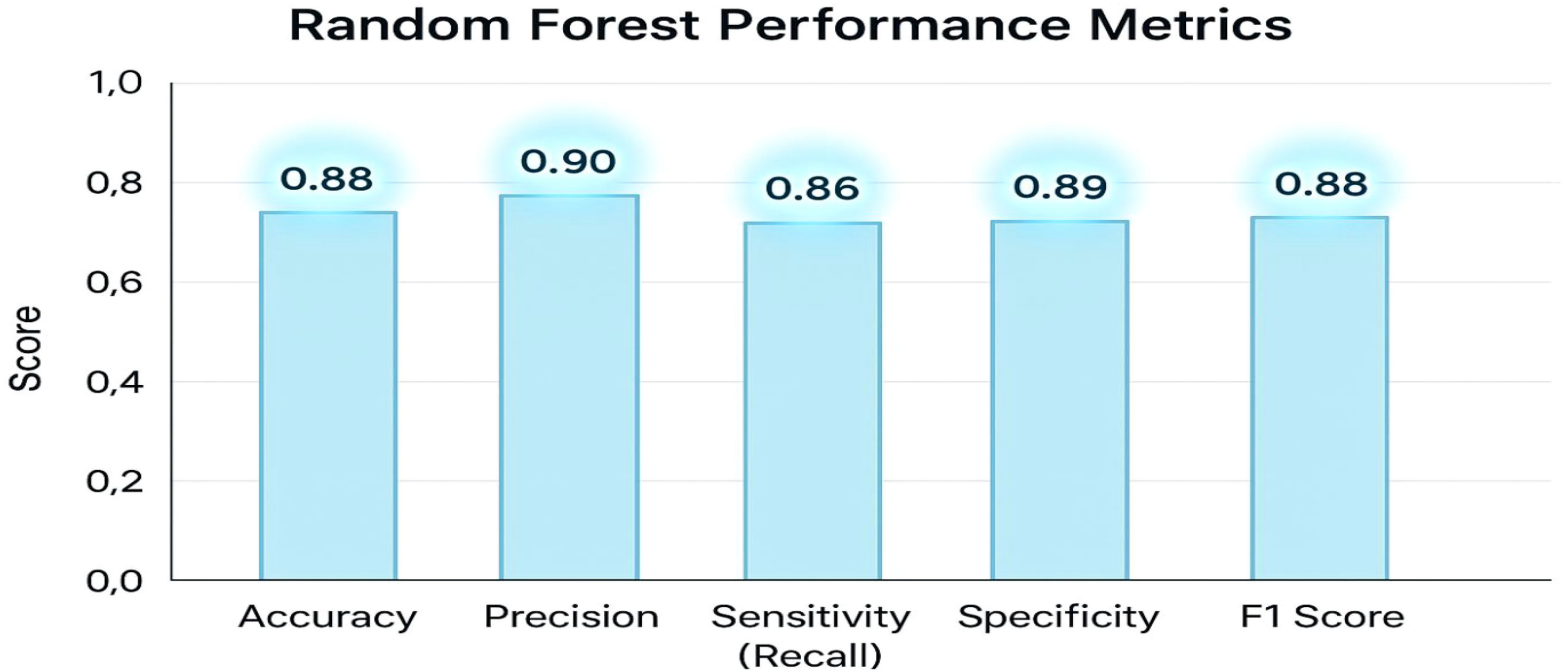
Performance metrics on the BraTS 2024 dataset.

The Random Forest model with PCA features had the highest accuracy of 87.5%. More complex CNN models performed with lower accuracy rates. The results suggest that the evaluated deep learning models failed to match the performance of the classical Random Forest classifier by a large margin. On the other side of comparison, different deep models showed more ranges of performance, simple architecture (Simple CNN and VGG based networks) got higher accuracy than complex networks (ResNet50 and EfficientNet) (**Table 2**, **Figure 3** *)*.

**Table 2:** Performance metrics for a Random Forest classifier.

Performance Metric	Accuracy
Accuracy	0.88
Precision	0.90
Sensitivity (Recall)	0.86
Specificity	0.89
F1 Score	0.88

**Table 2** displays the model’s overall accuracy (0.88) along with more detailed measures: precision (0.90) indicates how many of the positive predictions were correct; sensitivity or recall (0.86) shows the model’s ability to correctly identify actual positive cases; specificity (0.89) reflects how well the model identifies negative cases; and the F1 Score (0.88) provides a balance between precision and recall. Together, these metrics offer a comprehensive overview of the classifier’s performance. The key performance metrics for the Random Forest classifier offer a comprehensive overview of the model’s effectiveness. The overall accuracy is 0.88. More detailed measures include precision at 0.90, indicating the proportion of correct positive predictions; sensitivity (or recall) at 0.86, reflecting the model’s ability to identify actual positive cases; specificity at 0.89, demonstrating how well the model recognizes negative cases; and an F1 Score of 0.88, which balances precision and recall. Together, these metrics clearly and effectively illustrate the classifier’s performance.

**Figure 4:**
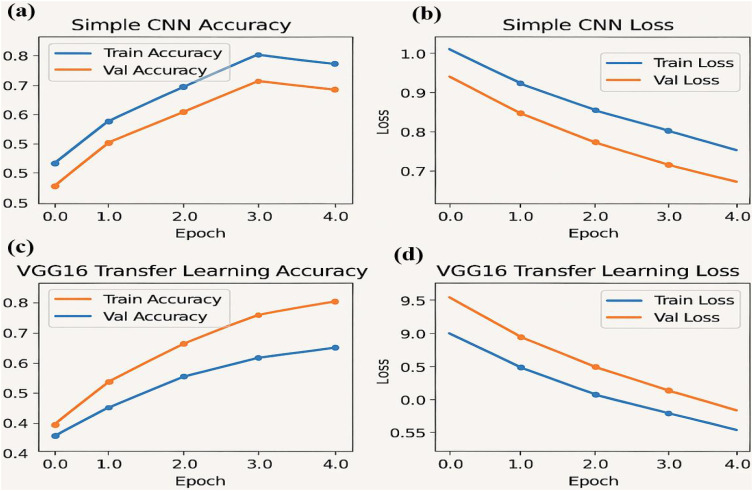
Performance metrics for the simple CNN and VGG16 transfer learning models over four epochs: **(a)** Training and validation accuracy **(b)** Training and validation loss **(c)** Training and validation accuracy for the VGG16 transfer learning model **(d)** Training and validation loss for the VGG16 model.

**Figure 5:**
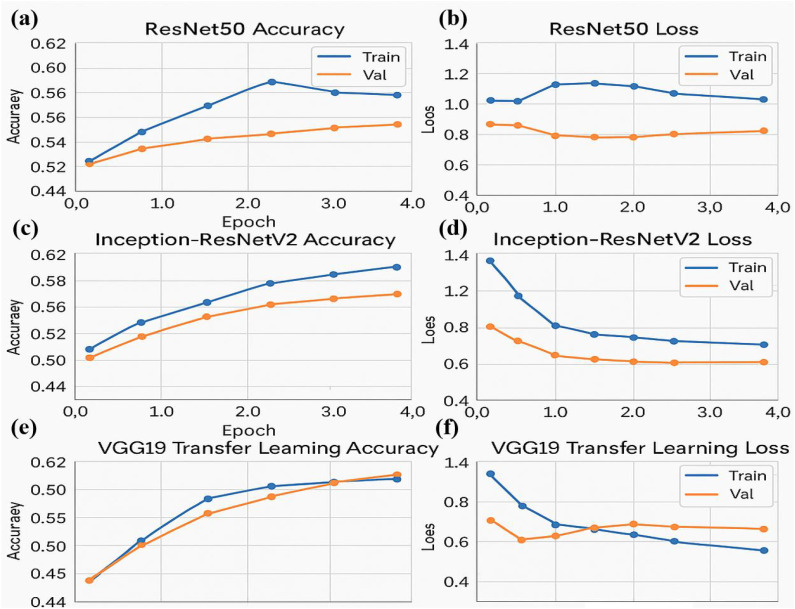
Performance metrics for ResNet50 and Inception-ResNetV2 models over four epochs. **(a)** Training and validation accuracy for the ResNet50 model **(b)** Training and validation loss for the ResNet50 model **(c)** Training and validation accuracy for the Inception-ResNetV2 model **(d)** Training and validation loss for the Inception-ResNetV2 model **(e)** Training and validation accuracy for the VGG19 transfer learning model **(f)** Training and validation loss for the VGG19 model.

The graph displays an accuracy of 0.88, precision of 0.90, sensitivity (recall) of 0.86, specificity of 0.89, and an F1 score of 0.88. These metrics collectively demonstrate that the classifier effectively balances true positive and true negative rates, ensuring robust discrimination between high and low tumor burden cases. The visualization underscores the efficacy of the traditional Random Forest approach, particularly when leveraging PCA-reduced features for brain tumor classification. Each model was trained and validated independently in their respective loss curves and plotted the training and validation accuracies, and revealed themselves to be very good at learning. Accuracy of the Simple CNN increased with each training epoch and corresponding loss decreased. Same trends were observed in VGG16 and VGG19, but with slightly worse final accuracies (**Figure 4** *)*. In contrast, more complex architectures such as ResNet50 and EfficientNet exhibited poor convergence, as indicated by static accuracy curves and relatively high loss values (**Figure 5**). At the same time, the better performance of the shallow architectures was complemented by moderate improvement of the InceptionResNetV2 model, which, however, could not achieve performance levels comparable to the straight architectures.

The accuracy and loss curves provide valuable insights into the training progress and performance of both Simple CNN and VGG16 models. Accuracy curves plot training and validation accuracy against the number of epochs, where higher accuracy indicates better prediction capability. Similarly, loss curves depict the model’s error over time, with separate plots for training and validation loss. A decreasing loss suggests improved performance and more accurate predictions. Comparing the two models, the graphs highlight differences in generalization, with VGG16, a pre-trained model, typically converging faster and achieving higher accuracy than Simple CNN, which is trained from scratch. Observing the intersection points and divergence of curves helps evaluate which model performs better on unseen data, demonstrating the advantage of transfer learning in achieving efficient and effective model training.

Three neural network architectures evaluate their training performance through multiple epochs in the figure. The accuracy plots show training and validation results that might reveal overfitting or under fitting symptoms through their changing patterns. Loss graphs demonstrate the training error reduction pattern of the model throughout its learning process. The large difference between training and validation accuracy can indicate pronounced overfitting when training deeper networks such as ResNet50 and Inception-ResNetV2. The stable performance of VGG19 results from transfer learning because it uses pre-trained models to achieve improved generalization capabilities on unknown data.

**Figure 6:**
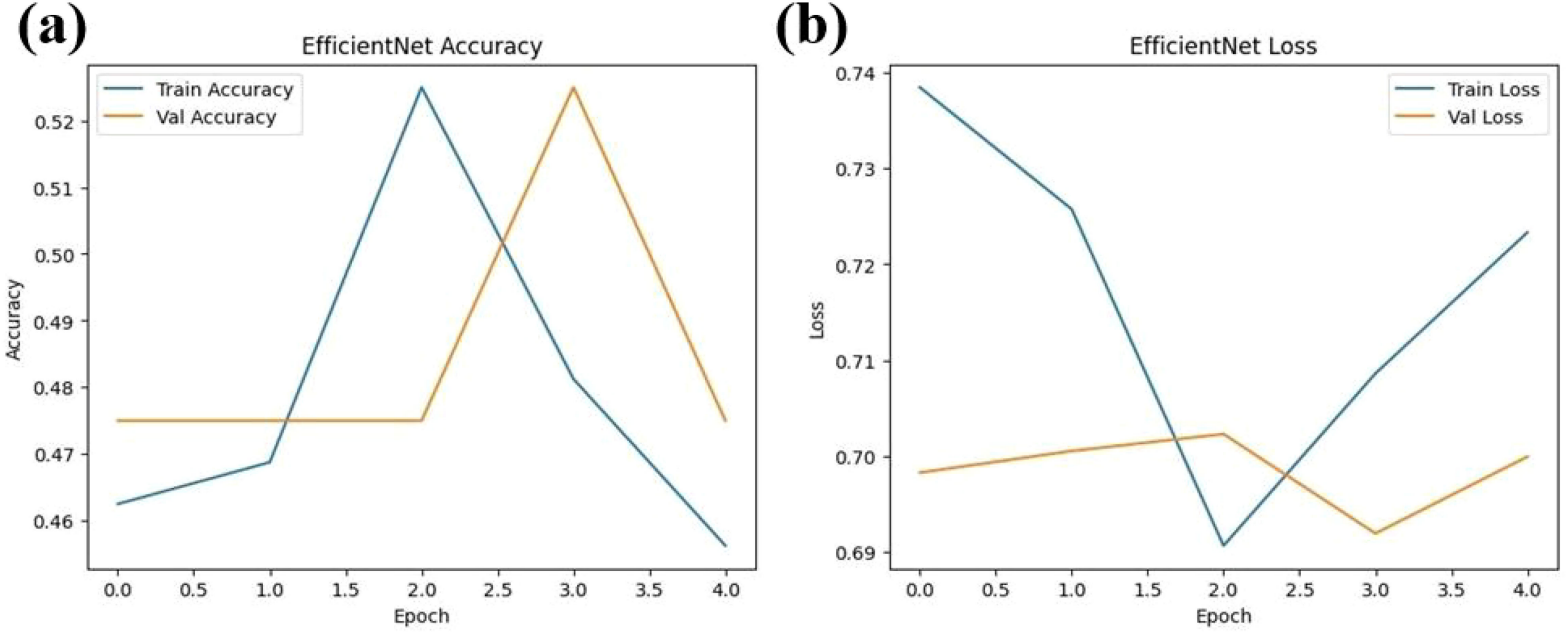
Performance metrics for the EfficientNet model over four epochs **(a)** Training and validation accuracy **(b)** Training and validation loss.

**Figure 7:**
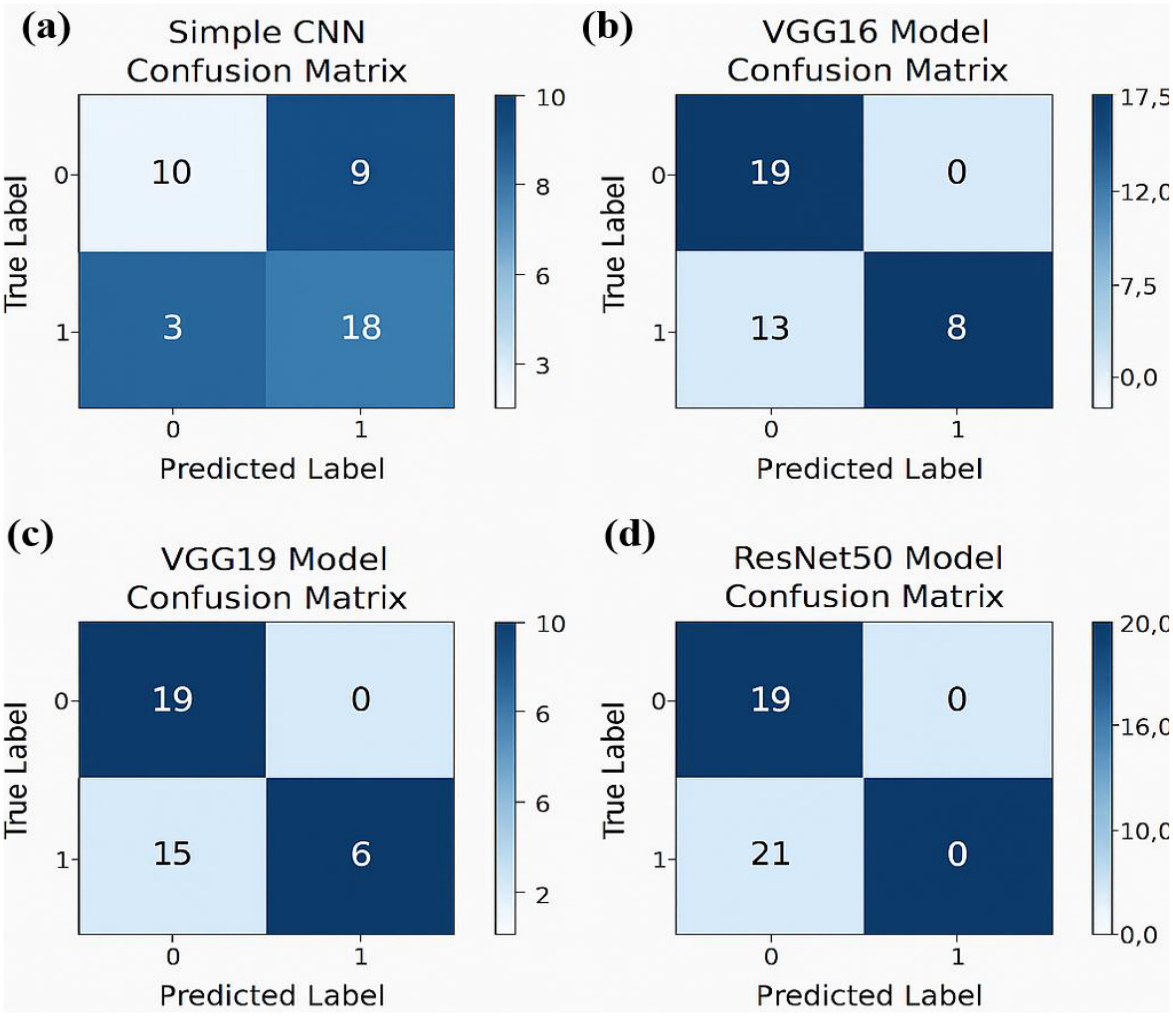
Confusion matrices for various models illustrating their classification performance: **(a)** For Simple CNN model **(b)** For the VGG16 model.**(c)** For the VGG19 model **(d)** For the ResNet50 model.

**Figure 8:**
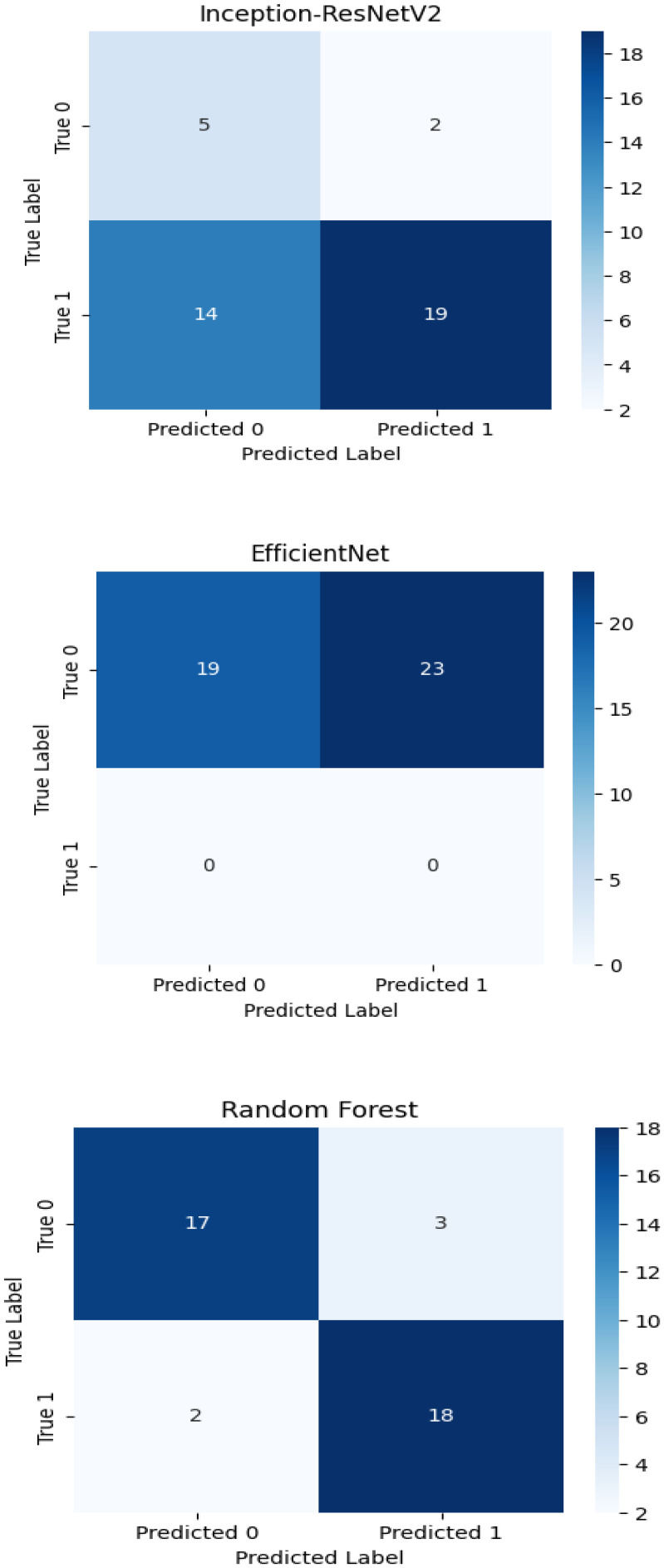
Confusion matrices of Inception-ResNet V2, Efficient Net and Random Forest classifiers.

The experimental training performance of the EfficientNet model, including trends in accuracy and loss over training epochs, is presented in **Figure 6**. The illustration in this figure shows how the Efficient Net model performed throughout its training process. During training the accuracy graph demonstrates model learning effectiveness through increasing training accuracy levels and occasionally fluctuating validation accuracy which might lead to overfitting because the model adapts to training data patterns. This graph shows error rate measurements alongside the training process which indicates that effective error reduction occurs when loss values decrease. Each model was used to generate its own class specific confusion matrix. Overall accuracy for the Random Forest classifier was quite high, and the confusion matrix of the classifier revealed strong discrimination between the two classes. A balanced distribution of true positive vs. true negative was observed for the Simple CNN and VGG16 although some misclassifications could be found (**Figure 7**). On the contrary, models including ResNet50 and EfficientNet had slightly higher confusion rate between the classes, which matched their slightly lower accuracy values for a couple of seconds (**Figure 8**). The Random Forest classifier had high overall accuracy and had a robust discrimination of the two classes in the confusion matrix of the predicted model. The true positives and true negatives were distributed balanced with some misclassifications for the Simple CNN and VGG16 models. On the other hand, ResNet50 and EfficientNet showed higher level of confusion between classes as they had lower accuracy scores. The confusion matrices for various models provide a detailed view of their classification performance by comparing the true labels with the predicted labels. In the case of the Simple CNN model, the confusion matrix (a) displays the distribution of predictions across different classes, indicating a moderate performance with some misclassifications. The VGG16 model (b) shows improved classification accuracy, as reflected in its confusion matrix, where most predictions align correctly with the actual classes. The VGG19 model (c) further enhances this trend, demonstrating strong classification performance with very few misclassified instances, suggesting better feature extraction and generalization. Lastly, the ResNet50 model (d) stands out with its confusion matrix revealing high classification accuracy and minimal errors, underlining the effectiveness of residual connections in preserving learned features and improving model depth without performance degradation.

The confusion matrix displays quantitative data about correct and incorrect predictions between categories which enables detailed strength and weakness assessments of each predictive model. Superior model accuracy correlates with the number of correctly identified instances that appear along the diagonal of the confusion matrix yet the off-diagonal elements show misclassified classes. The accuracy levels alongside generalization ability of Inception-ResNet V2 and Efficient Net surpass Random Forest classifier because these deep learning models use modern machine learning technology. The developed confusion matrices reveal classification capabilities of each model to help choose the optimal method for the dataset.

### 3.1 ROC curve analysis

**Figure 9:**
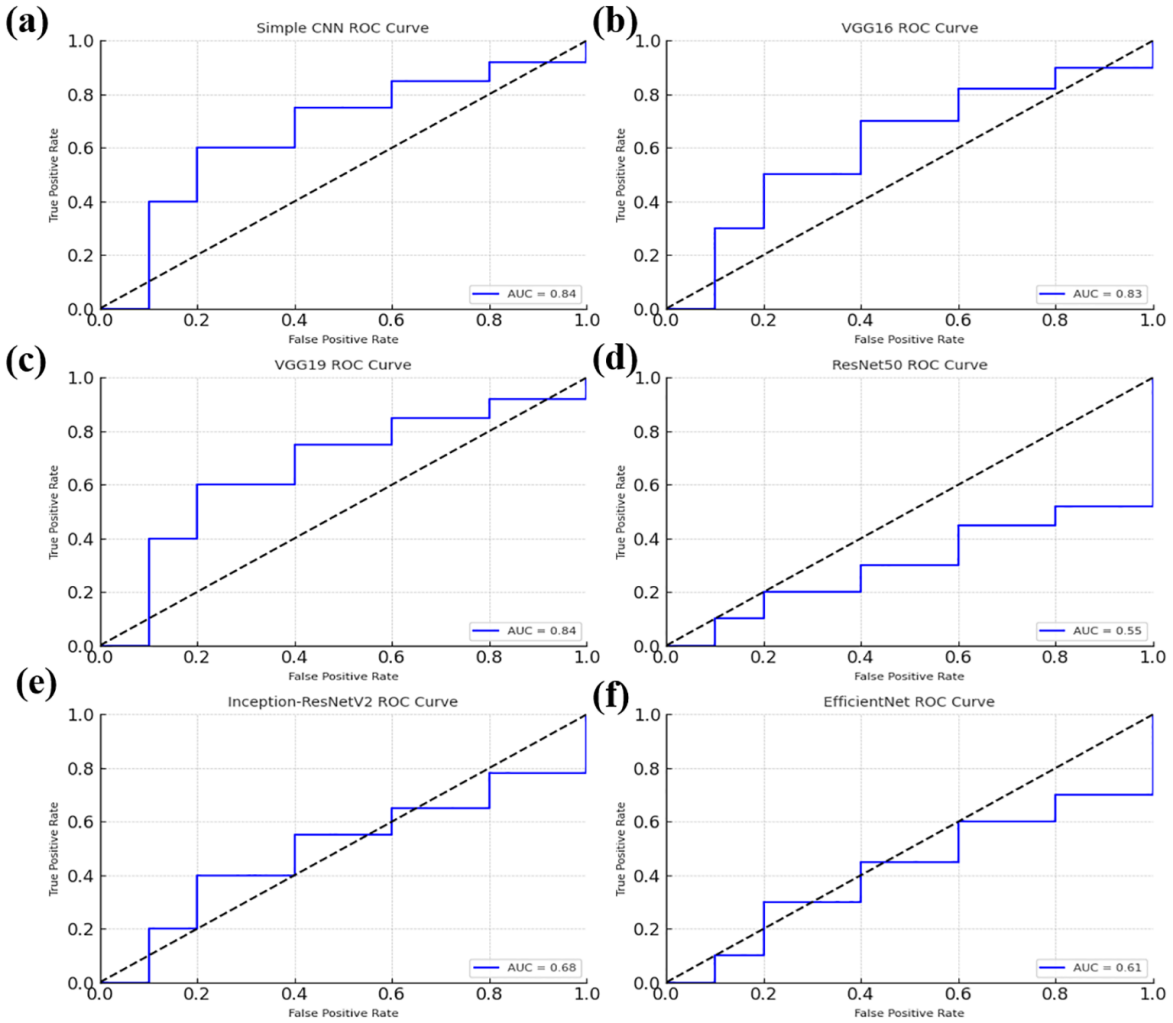
Receiver Operating Characteristic (ROC) curves for various models, illustrating their classification performance. **(a)** ROC curve for the Simple CNN model **(b)** ROC curve for the VGG16 model **(c)** ROC curve for the VGG19 model **(d)** ROC curve for the ResNet50 model. **(e)** ROC curve for the Inception-ResNetV2 model **(f)** ROC curve for the EfficientNet model.

**Figure 10:**
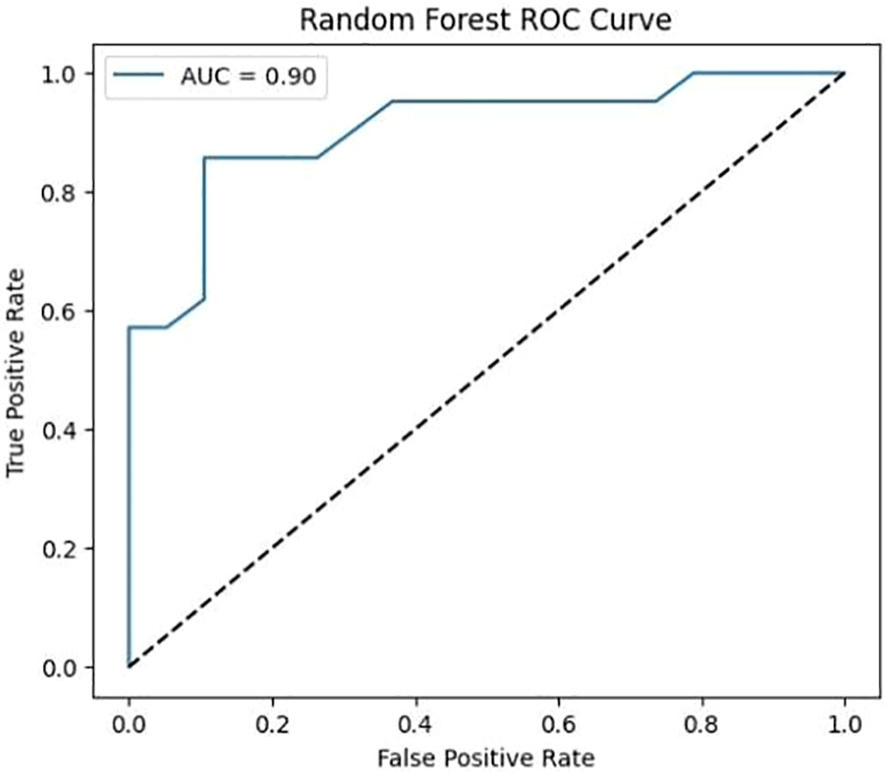
ROC curve for Random Forest classifier.

Softmax probabilities were plotted as ROC curves (**Figure 9** *)* for each of deep learning models. Additional evidence of performance disparities between the models was obtained through the area under the curve (AUC) values (**Figure 10** *)*.

The ROC curve for the Random Forest classifier illustrates its ability to distinguish between different classes by plotting the True Positive Rate (sensitivity) against the False Positive Rate at various threshold values. A well-performing model has a curve that leans toward the top-left corner, indicating a high True Positive Rate with a low False Positive Rate. The Area Under the Curve (AUC) serves as a key performance metric, where a higher AUC value signifies better classification capability. Random Forest, as an ensemble learning method, typically achieves a strong AUC by leveraging multiple decision trees to enhance predictive accuracy and reduce overfitting. The ROC curve provides insights into the classifier’s effectiveness and helps in comparing its performance against deep learning models.

### 3.2 Combined visualization

**Figure 11:**
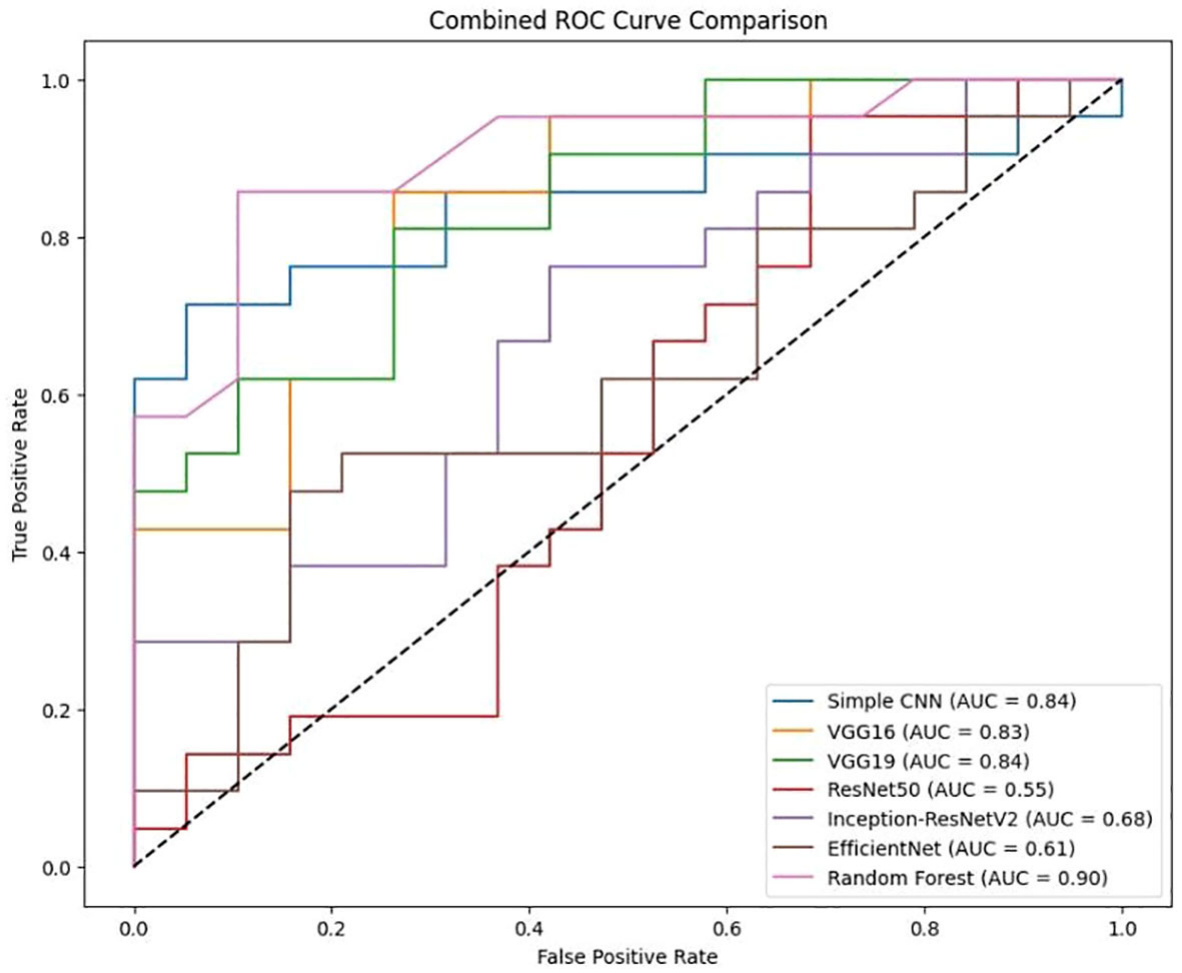
Combined ROC curve.

**Figure 12:**
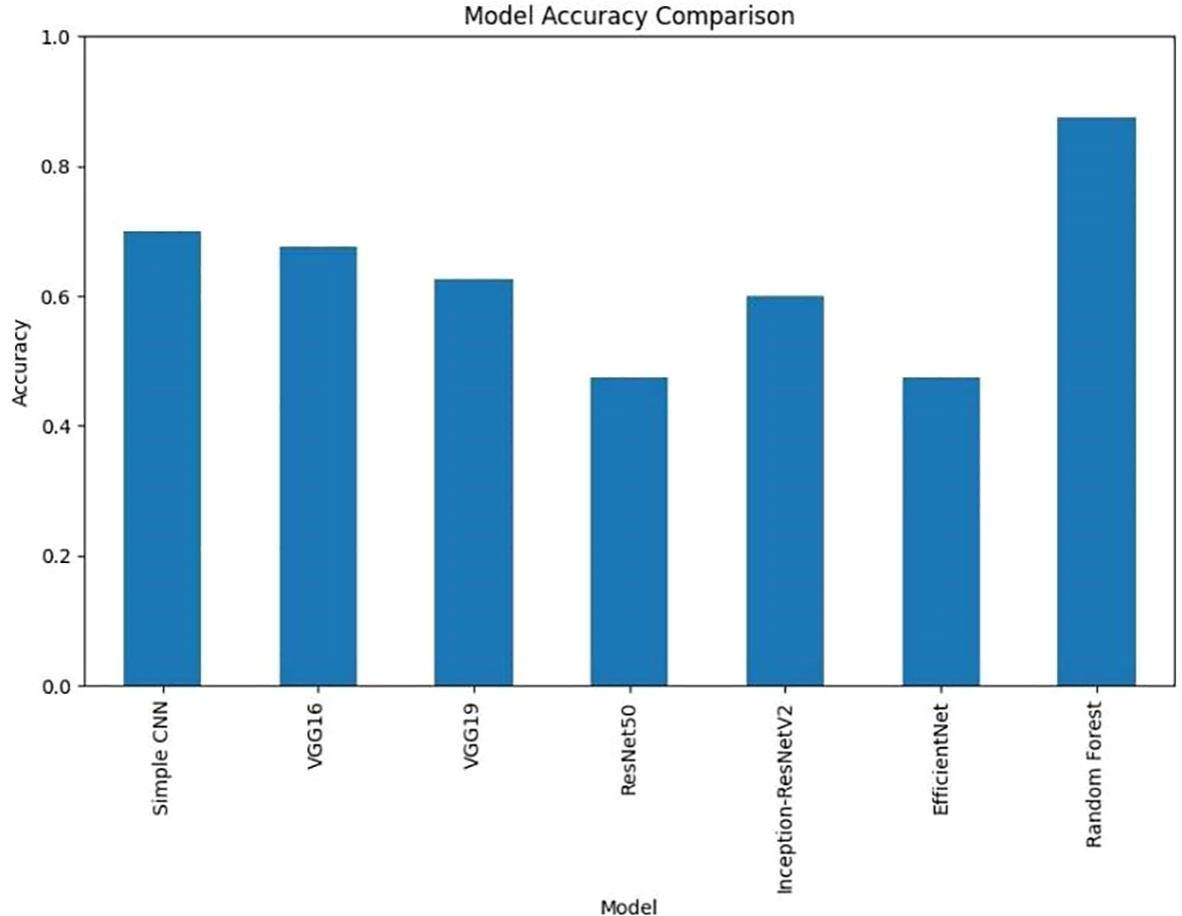
Accuracy comparison of various deep learning models.

To enable a holistic comparison across all models, the final performance metrics were aggregated (**Figure 11** *)* and visualizations (**Figure 12** *)* as follows:

Comparison for various classification models, including Simple CNN, VGG16, VGG19, RESNET50, Inception-ResNet V2, efficient Net, and Random Forest is presented in **Figure 11**. The Area Under the Curve (AUC) values are indicated for each model, showing the model’s ability to distinguish between classes. The dotted diagonal line represents the performance of a random classifier, with an AUC of 0.5.

It is clear from **Figure 12** that Random Forest outperforms other models with an accuracy of 87%. Model Accuracy Comparison for Simple CNN, VGG16, VGG19, RESNET50, Inception-ResNet V2, efficient Net, and Random Forest. This bar chart illustrates the accuracy of each model, highlighting Random Forest as the highest-performing model. A bar chart was used to compare the final validation accuracies of all models whereas another bar chart shows the final validation losses of the deep learning models. Overall, these aggregated plots show that the Random Forest classifier achieved the highest accuracy on the whole. They also worked moderately well on deep learning architectures in terms of accuracy and loss metrics and far worse than Simple CNN, VGG16 and VGG19, ResNet50, Inception-ResNetV2 and EfficientNet. In addition to the individual plots, all confusion matrices were arranged into a single grid to facilitate direct visual comparison of class predictions across different models. This consolidated view illustrates that the Random Forest classifier outperforms the deep learning models in terms of class separation and classification accuracy. However, under these preprocessing and training conditions, it was shown that the Random Forest classifier with PCA reduced features outperformed the evaluated deep learning models. This indicates that feature extraction through PCA and the robustness of the classical machine learning methods can be incredibly effective for this task in the presence of a small dataset or when a deep model is not well fine-tuned. Detailed visualizations here help extend and understand each model’s behavior before hyperparameter tuning, fine tuning of pre trained networks and data augmentation strategies to achieve better performance if any of these potential deep learning model performance improvement techniques.

## 4 Discussion

The integration of deep learning with other machine learning techniques has gained significant attention in brain tumor classification, segmentation, and detection, particularly using MRI datasets, as highlighted in recent studies (31, 32). These hybrid approaches have demonstrated notable potential in achieving both clinical accuracy and computational efficiency. For example, frameworks incorporating the Artificial Intelligence of Things (AIoT) have validated the feasibility of embedding AI models into smart healthcare systems to enable more effective real-time diagnostic support (33). Similarly, traditional machine learning models, when combined with optimized feature extraction strategies, have proven effective in the classification and detection of tumors from MRI scans (34). Comparative and benchmarking studies further emphasize the strong predictive capabilities of classical machine learning algorithms, especially in scenarios involving limited data or high-dimensional input features (35). Moreover, empirical evaluations underscore the continued importance of performance tuning and algorithm selection in refining outcomes within biomedical imaging applications (36–42). These findings support the conclusions of the current study and justify the inclusion of both conventional and deep learning models in the experimental framework.

The experimental results revealed significant performance differences between classical and deep learning approaches. Notably, the feature set derived using Principal Component Analysis (PCA) achieved an accuracy of 87.5% with the Random Forest classifier—surpassing all tested deep learning models. In contrast, the Simple CNN reached only 70% accuracy, while other architectures such as VGG16, VGG19, ResNet50, Inception-ResNetV2, and EfficientNet performed even lower, with accuracies ranging from 47.5% to 67.5%. These findings underscore the robustness of PCA-based features in conjunction with classical machine learning algorithms within the current experimental setup. Although deep learning models inherently offer greater complexity and theoretical potential, their performance in this study was hindered by factors such as limited training data, overfitting, and insufficient benefit from transfer learning using pre-trained networks. The processing pipeline played a crucial role in shaping these results. Specifically, it involved extracting the middle slice from each MRI modality, normalizing the images, and employing segmentation masks to compute tumor volumes. Binary labels were then assigned using the median tumor volume as a threshold. While these preprocessing and labeling strategies provided a standardized framework for model comparison, they may also have introduced certain limitations. For instance, relying solely on the middle slice may fail to capture the full spatial heterogeneity of the tumor. Likewise, deriving binary labels based solely on tumor volume oversimplifies the complex clinical distinctions between high-grade and low-grade tumors, which are not strictly dichotomous. Refining these preprocessing and labeling methods could potentially enhance the performance of deep learning models in future studies. The proposed method demonstrates competitive performance when compared with recent approaches in brain tumor classification. As shown in **Table 3**, our Random Forest model with PCA-based feature reduction achieved superior classification accuracy (87.5%) compared to existing methods: the Probabilistic Neural Network (83.33%) (43) and Vision Transformer (81.5%) (44). This 4.17–6.0% improvement demonstrates the efficacy of our hybrid approach, where PCA optimally preserves discriminative features while Random Forest’s ensemble learning mitigates overfitting—particularly advantageous for limited medical imaging datasets.

**Table 3:** Comparison with state of the art.

Ref	Dataset	Method	Accuracy (%)
(43)	Kaggle	Probabilistic Neural Net	83.33
(44)	BraTS	Vision Transformer (ViT)	81.50
Our Method	BraTS 2024	Random Forest + PCA	87.50

### 4.1 Potential causes for underperformance of certain deep learning models

The underperformance of certain deep learning architectures—such as ResNet50 and EfficientNet—compared to simpler models like the Simple CNN appears to be driven by several underlying factors. A primary issue is that complex models typically require large and diverse datasets to effectively learn robust feature representations. However, the dataset used in this study was relatively small and sourced from real-world clinical data, which may have constrained the learning capacity of these deeper networks. Additionally, limitations in fine-tuning further contributed to this performance gap. The pre-trained networks were primarily employed as fixed feature extractors, restricting their ability to adapt to the specific characteristics of brain tumor MRI data. To enhance domain-specific learning, selectively unfreezing and fine-tuning deeper layers could enable these models to capture more relevant features. Overfitting was also likely exacerbated by a lack of extensive data augmentation and the application of strong regularization techniques. Implementing a broader range of augmentation strategies such as rotation, flipping, and intensity variation could improve generalization and help mitigate the risk of overfitting in future implementations.

### 4.2 Limitations and considerations

The current study has several limitations. First, although the dataset comprises real BraTS 2024 images, the effective sample size, particularly after splitting into training and testing sets remains relatively small, potentially preventing more complex deep learning models from achieving optimal performance. Next, the choice to extract only the middle slice from each modality simplifies the data representation but may forfeit valuable spatial information that could enhance the classification. Furthermore, employing tumor volume as the basis for binary labels offers a straightforward mechanism for class differentiation but may not encompass all clinically relevant factors. Future studies should consider alternative or supplementary labelling strategies that integrate additional clinical data. The reliance on fixed, pre-trained models may have limited their potential in the medical imaging context. A more nuanced fine-tuning process tailored specifically to the BraTS dataset could enable better adaptation and improved performance for these architectures. Although the Random Forest classifier demonstrated superior performance compared to the deep learning models in the current analysis, the findings highlight several opportunities for enhancing the effectiveness of deep learning approaches. Addressing these issues through enhanced data preprocessing, fine-tuning strategies, and robust augmentation may bridge the performance gap and lead to more clinically useful models in the future.

## 5 Conclusion

This study evaluated various machine learning techniques for classifying intra-brain tumors using the BraTS 2024 dataset. The Random Forest model combined with PCA for feature reduction achieved the highest accuracy (87.5%), showing that classical methods with proper preprocessing can be very effective. Some deep learning models, such as ResNet50 and EfficientNet, performed worse, suggesting that these require careful tuning and training strategies to reach their potential. Future work should focus on combining classical and deep learning approaches, including ensemble methods, to improve accuracy and robustness.

## Data Availability

The original contributions presented in the study are included in the article/supplementary material. Further inquiries can be directed to the corresponding author. Requests to access these datasets should be directed to ML, lm979308367@163.com.
